# FBXL6 governs c-MYC to promote hepatocellular carcinoma through ubiquitination and stabilization of HSP90AA1

**DOI:** 10.1186/s12964-020-00604-y

**Published:** 2020-06-23

**Authors:** Weidong Shi, Lanyun Feng, Shu Dong, Zhouyu Ning, Yongqiang Hua, Luming Liu, Zhen Chen, Zhiqiang Meng

**Affiliations:** 1 Department of Integrative Oncology, Fudan University Shanghai Cancer Center, 270 Dong An Road, Shanghai, 200032 China; 2 Department of Oncology, Shanghai Medical College, Fudan University, Shanghai, China; 3 Collaborative Innovation Center for Cancer Medicine, Fudan University Shanghai Cancer Center, Shanghai, China

**Keywords:** Hepatocellular carcinoma, Heat shock proteins, HSP90AA1, SCF complex, C-MYC, FBXL6, Ubiquitination, F-box protein, E3 ligase, Degradation

## Abstract

**Background:**

Heat shot protein 90 (HSP90) AA1 functions as an onco-protein to regulate the assembly, manipulation, folding and degradation of its client proteins, including c-MYC. However, little is known about the mechanism of HSP90AA1 regulation.

**Methods:**

Transcriptome RNA-sequencing data of hepatocellular carcinoma (HCC) samples were used to detect the mRNA expression of FBXL6. Immunoprecipitation/Mass Spectrum (IP/MS) method was used to identify the interacting proteins of FBXL6. The co-immunoprecipitation assay was used to determine the interaction between FBXL6 and HSP90AA1. The in vivo ubiquitination assay was performed to determine the regulation of HSP90AA1 by FBXL6. Luciferase reporter and chromatin immunoprecipitation (ChIP) assays were used to determine the transcriptional regulation of FBXL6 by c-MYC. Immunohistochemical (IHC) staining was performed to study the correlation of FBXL6 and HSP90AA1 protein expression in 87 HCC samples. Cell counting and colony formation assays were implemented to detect the biological effects of FBXL6 on the growth of HCC cells in vitro. The effect of FBXL6 on HCC tumor growth in vivo was studied in a tumor xenograft model in mice.

**Results:**

Here, we identified the orphan F-box protein FBXL6, a substrate recognition subunit of an SCF (Skp1-Cul1-F-box protein) complex, as the ubiquitin ligase for HSP90AA1. FBXL6 promoted K63-dependent ubiquitination of HSP90AA1 to stabilize it. Through analysis of the TCGA dataset, we found that FBXL6 was significantly increased in HCC tissues and positively correlated with c-MYC pathway. FBXL6 accumulation in HCC causes the stabilization and activation of c-MYC by preventing HSP90AA1 degradation. The activated c-MYC directly binds to the promoter region of FBXL6 to induce its mRNA expression.

**Conclusion:**

Collectively, our data revealed an unknown FBXL6-HSP90AA1-c-MYC axis which might contribute to the oncogenesis of HCC, and we propose that inhibition of FBXL6 might represent an effective therapeutic strategy for HCC treatment.

Video abstract

## Background

Hepatocellular carcinoma (HCC) is one of the most common cancer in the world and the second malignant tumor of global cancer mortality, and its morbidity and mortality are gradually increasing [1]. The pathogenesis of HCC is extremely complicate and the discovery of new molecular drug targets will benefit HCC treatment [2].

The ubiquitin (Ub)-proteasome system (UPS) plays a prominent role in a variety of cellular activities, including cell cycle control, apoptosis, DNA damage repair, immune response and tumorigenesis [3]. Ubiquitination is catalyzed by a three-enzyme cascade consisting of the E1 Ub-activating enzyme, the E2 Ub-conjugating enzyme, and the E3 Ub-protein ligase [4]. In UPS, Ub modifies protein substrates mostly in the form of a K48-or K11-linked polyUb chain, which serves as a signal for proteasome-dependent degradation [5]. However, K63-linked polyUb chain is not associated with proteasome degradation of the substrate protein [6].

The selectivity of Ub-mediated proteolysis is determined by the E3 ligases which could be grouped into two classes based on their structural features: the RING (really interesting new gene) E3s and the HECT (homologous to the E6AP carboxyl terminus) E3s. The RING E3s constitute the largest E3 ligases family with more than 600 documented members, which directly catalyze the transfer of ubiquitin from an E2 to a substrate [7]. The substrates-recruiting and catalytic modules could be found in a single polypeptide or in different subunits of a E3 complex, such as the anaphase-promoting complex (APC) and the Cullin–RING ligases (CRLs) [8]. In mammals, there are approximately 200 CRLs has been reported. Each of CRLs contains a different Cullin subunit that using its carboxyl terminus to bind to the E2 enzyme and N terminus to bind to the substrate recognition factors. The CRL1 ligases, better known the SCF (Skp1-Cul1-F-box protein) complex, are the best characterized. SCF is a four-protein complex consisting of the constant Cullin1, RBX1, SKP1 and one of ~ 70 various F-box proteins [9]. Early studies have demonstrated that F-box proteins play indispensable roles in cell cycle regulation [10–12], and in recent years more and more F-box proteins have been reported to be closely related to tumorigenesis [13, 14]. However, given the larger number of F-box proteins, only a few F-box proteins have identified substrates and functions.

Heat shock proteins (HSPs) are a class of highly conserved proteins during biological evolution and widely found in prokaryotic and eukaryotic organisms [15]. HSPs could be induced under diverse stress conditions (virus infection, hypoxia, ultraviolet radiation, etc.). HSPs are both biomarkers of cellular stress response and also important molecular chaperone proteins in cells [15]. HSPs participate in maintaining the correct folding of the client’s protein, enabling the protein to form the conformation required for physiological functions, thereby playing an important role in regulating the balance of protein synthesis/degradation and protein localization [16]. HSPs are mainly divided into five families: HSP90 family (83–90 kD), HSP70 family (66–78 kD), HSP60 family, small molecule smHSP family (15–30 kD), and macromolecular HSPs with molecular weights ranging from 100 to 110 kD. Among them, HSP90 is abundant in cells, accounting for 1 to 2% of total cellular protein [17]. In humans, there are four Hsp90 isoforms including the cytoplasmic Hsp90α and Hsp90β, as well as the endoplasmic reticulum isoform Grp94 and mitochondrial isoform TRAP1, respectively [18]. Hsp90α (Hsp90AA1), encoded by the HSP90aa1 gene, is composed of three major domains: the N-terminal domain, the intermediate domain, and the C-terminal domain. These three domains work together to play the molecular chaperone function of HSP90AA1, which is dependent on the binding of ATP to the ATPase domain at the N-terminus [19]. The binding and hydrolysis of ATP produces a conformational transition that regulates the assembly of the multi-subunit complexes involved. HSP90AA1 plays an important role in the assembly, manipulation, folding and degradation of its client proteins. Numerous studies have shown that inhibition of HSP90AA1 function can lead to degradation of its client protein through the ubiquitin-protease pathway [20]. Many of the client proteins regulated by HSP90AA1 are proto-oncogene products (such as c-MYC) or important signal transduction factors during tumor pathogenesis, which are closely related to the occurrence and development of tumors [21, 22]. Thus, inhibition of HSP90AA1 might affect cancer cells growth and survival from multiple pathways, making HSP90AA1 a promising anti-tumor drug target.

Here, through analysis of the TCGA dataset, we found that the mRNA expression of an orphan F-box protein FBXL6 was significantly increased in HCC compared with normal tissues and positively correlated with c-MYC expression. We further showed that FBXL6 forms a classical SCF E3 ligase complex to exert its oncogenic roles by stabilizing HSP90AA1 to activate c-MYC, which in turn directly bound to the promoter region of FBXL6 to induce its expression. Thus, our data revealed an unknown positive feedback axis of FBXL6-HSP90AA1-c-MYC, whose abnormal activation might contribute to the oncogenesis of HCC.

## Material and methods

### Clinical samples and data acquisition

Transcriptome RNA-sequencing data of hepatocellular carcinoma (HCC) samples were downloaded from the TCGA data portal (https://cancergenome.nih.gov/), which contained data from 374 primary HCC and 50 non-tumor tissues. Raw count data was downloaded for further analyses. To selected genes involved in the onset of HCC, differentially expressed genes between HCC and non-tumor tissues were screened via the R software Linear Models for Microarray and RNA-Seq Data (Limma) package (http://bioconductor.org/packages/Limma/). We performed differential gene analysis of all transcriptional data, setting a log2 |fold change| > 1 and a false discovery rate (FDR) < 0.05 as the cutoff values. The Wilcox-test was used for analyses.

### Immunohistochemical (IHC) staining

The HCC cancer tissues and matched adjacent tissues (at least 2 cm from the surgical incision) were collected from 87 patients with hepatocellular carcinoma who were surgically resected at Fudan University Shanghai Cancer Center from January 2016 to December 2018. All specimens were confirmed by pathological diagnosis. No patients received radiotherapy or chemotherapy before surgery. These tissues were placed in liquid nitrogen and then transported to − 80 °C refrigerator for storage. Written informed consent was obtained from each patient before sample collection, and the study protocol was approved by the Medical Ethics Committee of Fudan University Shanghai Cancer Center. These 87 HCC clinical samples were fixed in 4% paraformaldehyde (PFA), embedded in paraffin, sectioned and stained with haematoxylin and eosin. IHC staining of the paraffin-embedded tumor tissues was performed using anti-FBXL6 and anti-HSP90AA1 antibodies. We have added these information in the revised manuscript.

### Cell culture and reagents

HEK293T cells and hepatocellular carcinoma cell lines SMMC-7721 and Hep3B cells were purchased from American Type Culture Collection (ATCC). Cells were cultured in Dulbecco’s modified Eagle’s medium (DMEM) (Invitrogen), supplemented with 10% FBS (Gibco), 100 units/mL penicillin, and 100 mg/mL streptomycin (Gibco). MG132 and Cycloheximide (CHX) were purchased from Sigma.

### Plasmids

F-box protein genes were amplified from 293 T or SMMC-7721 cells by polymerase chain reaction and cloned into pbabe-Flag vector. pCherry.90 alpha was a gift from Didier Picard (Addgene plasmid # 108222; http://n2t.net/addgene:108222; RRID:Addgene_108,222). c-myc-PT3EF1a was a gift from Xin Chen (Addgene plasmid # 92046; http://n2t.net/addgene:92046; RRID:Addgene_92,046). pRK5-HA-Ubiquitin-K63 was a gift from Ted Dawson (Addgene plasmid # 17606; http://n2t.net/addgene:17606; RRID:Addgene_17,606). All plasmids were completely sequenced and transfected into cells by using Lipofectamine 2000 (Invitrogen) according to manufacturer’s instructions.

### RNA interference, RNA isolation and real-time PCR

The Lentiviral Human FBXL6 shRNA was purchased from Merck and the target sequences for short hairpin RNA (sh-RNA)-expressing plasmids were the following: FBXL6-shRNA1: CACCGGCATCAACCGTAATAG; FBXL6-shRNA2: TGGAGTGGCTTATGCCCAATC. Total RNA of cell lysate was extracted by using TRIzol reagent (Invitrogen, Shanghai). Oligo dT was used to prime cDNA synthesis. Real-time PCR was then performed by using a SYBR Green Premix Ex Taq (TaKaRa) on Light Cycler480 (Roche, Switzerland). GAPDH was used as internal control. Differences in gene expression were calculated using 2-ΔΔCt method. Primers used for qPCR analysis were list as follows: FBXL6 forward, 5′- GGAGACCGCATTCCCTTGG-3′; reverse, 5′- AAAACCGATTGGGCATAAGCC-3′. HSP90AA1 forward, 5′- AGGAGGTTGAGACGTTCGC-3′; reverse, 5′- AGAGTTCGATCTTGTTTGTTCGG-3′. GAPDH forward, 5′- TGTGGGCATCAATGGATTTGG − 3′; reverse, 5′- ACACCATGTATTCCGGGTCAAT -3′.

### CRISPR/Cas9 knock out (KO) cell lines

SMMC-7721 cells were transfected with FBXL6 CRISPR/Cas9 KO (h) KO plasmid (sc-408,853, Santa Cruz Biotechnology) using Lipofectamine2000 following the manufacturer’s instructions. Cells were selected with 1 μg/ml puromycin 2 weeks. Single clones were then selected and the knockout efficiency was verified by western blot assay.

### Western blotting and antibodies

Cells were lysed with lysis buffer (100 mM Tris-HCl, pH 6.8, 100 mM DTT, 1% SDS, 10% glycerol). Proteins were separated by 10–12% SDS-PAGE, and transferred to NC membrane. Membranes were blocked in 5% non-fat milk in phosphate-buffered saline (PBS) for 1 h before incubation with primary antibody overnight at 4 °C. Membranes were washed with and incubated with secondary antibody for 1 h. Primary antibodies used as indicated: anti-Flag M2 (1:4000 dilution, F1804, Sigma), anti-HSP90AA1 (1:1000 dilution, 13,171–1-AP, Protein tech), anti-FBXL6 (1:1000 dilution, SAB1407299, Sigma), anti-Cul1 (1:1000 dilution, sc-17,775, Santa cruz, U.S.A), anti-SKP1 (1:2000 dilution, #12248, Cell Signaling Technology, U.S.A), anti-c-Myc (1:1000 dilution, #18583, Cell Signaling Technology, U.S.A), and anti-GAPDH (1:5000 dilution, #5174, Cell Signaling Technology, U.S.A).

### Immunoprecipitation (IP) and mass spectrometry (MS)

Cells were lysed with IP buffer (100 mM NaCl, 20 mM Tris-cl PH8.0, 0.5 mM EDTA, 0.5% (v/v) Nonidet P-40) with protease inhibitor cocktail and phosphorylate inhibitor for 30 min on ice. Cells were sonicated and the lysates were centrifuged. The supernatant was incubated with appropriate antibodies and protein A/G beads overnight at 4 °C in a rotating wheel. Immunoprecipitates were washed with IP buffer. SDS loading buffer was then added and proteins were eluted by boiling at 95°Cfor 5 min. For mass spectrometry assay, lysates from 293 T cells transfected with Flag-con or Flag-FBXL6 were cleared by centrifugation at 15,000 g for 20 min at 4 °C to remove cell debris. The resulting lysates were subjected to IP with Flag M2 beads overnight at 4 °C. Bound proteins were eluted by boiling, resolved by SDS-PAGE and stained with coomassie blue staining, followed by mass spectrometry analysis.

### In vivo Ubiquitination assay

Cells co-transfected His-K63-Ubiquitin with EV or Flag-FBXL6 plasmids were sonicated in IP buffer containing 8 M urea and 10 mM imidazole. His-K63-Ubiquitin-conjugated proteins were recovered with Ni-NTA resin (Qiagen), washed eight times in urea lysis buffer containing 20 mM imidazole, and eluted with IP buffer containing 5% SDS and 200 mM imidazole. The boiled samples were separated by 10% SDS–PAGE and subjected to western blot with antibodies as indicated. For endogenous ubiquitinated protein accumulation, Tandem Ubiquitin Binding Entity 2 (TUBE2) resin (LifeSensors) was used. Cells were lysed with IP buffer with protease inhibitor cocktail and phosphorylate inhibitor for 30 min on ice. Cells were sonicated and the lysates were centrifuged. The supernatant was incubated with TUBE2 resin overnight at 4 °C in a rotating wheel. The resin was then washed with IP buffer and boiled in SDS loading buffer. Boiled samples were separated by 10% SDS–PAGE and subjected to western blot with antibodies as indicated.

### Colony formation analysis

Cells were seeded in a six-well plate at a density of 1000/well and then cultured for 2 weeks. The numbers of colonies containing more than 50 cells were counted by crystal purple staining.

### Apoptosis analysis

Cells were seeded into 6 well plates. Apoptosis cells were determined using Annexin V–fluorescein isothiocyanate (FITC) and propidium iodide (PI) apoptosis detection kit according to the manufacturer’s instruction. Cell apoptosis was then analyzed using a FACS Calibur flow cytometer (BD Biosciences, San Jose, CA, USA). Apoptosis was also determined by measuring the activity of the caspases 3 and 7 using a luminescent substrate (Caspase-Glo 3/7; Promega) according to manufacturer’s instructions.

### Luciferase reporter and chromatin immunoprecipitation assays

The promoter region of FBXL6 gene was amplified from the human genomic DNA and inserted into pGL4.15 vector (Promega, Madison, Wisconsin, USA). For the luciferase reporter assays, HEK293T cells were seeded in 24-well plates and transfected with the indicated plasmids using Lipofectamine 2000 (Invitrogen) for 36 h. Luciferase activity was measured using the Dual Luciferase Reporter Assay System (Promega). The firefly luciferase luminescence data were normalized by the Renilla luciferase luminescence data. A chromatin immunoprecipitation (ChIP) assay kit (Upstate, Billerica, MA) was used according to manufacturer instructions. Briefly, cells were fixed with formaldehyde and DNA was sheared to fragments at 200–1000 bp by repeated sonication. Chromatin was then incubated and precipitated with antibodies against c-Myc or IgG. Primers for GAPDH were used as negative control.

### Xenograft assays

Animal study was approved by Animal Care and Use Committee of Fudan University Shanghai Cancer Center. 8-week-old male BALB/cA nude mice were purchased from National Rodent Laboratory Animal Resources (Shanghai, China). All mice were kept in a specific pathogen-free facility and housed at 21 °C ± 1 °C with humidity of 55 ± 10%, fed with sterilized food and water, and kept on a 12 h light/dark cycle. FBXL6^+/+^and FBXL6^−/−^ SMMC-7721 cells at a density of 1 × 10^7^ were suspended in 50 μl of DMEM medium, mixed 1:1 with Matrigel (Corning) and injected into the flanks of male nude mice. Tumor sizes were measured by a caliper and calculated using the formula length × width 2 × 1/2. Tumor weights were measured after mice were sacrificed.

### Statistical analyses

All experiments were at least repeated three times. Data are presented as mean ± standard deviation (SD). Statistical analysis was performed with GraphPad Prism 7.0 software. The differences between groups were calculated using the Student’s t-test or one-way ANOVA using a Tukey post-hoc test. *P* values of < 0.05 were considered statistically significant. Statistical significance is displayed as * *P* < 0.05, ** *P* < 0.01, and *** *P* < 0.001, respectively.

## Results

### FBXL6 is highly expressed in HCC and associated with the c-MYC pathway

**Fig. 1:**
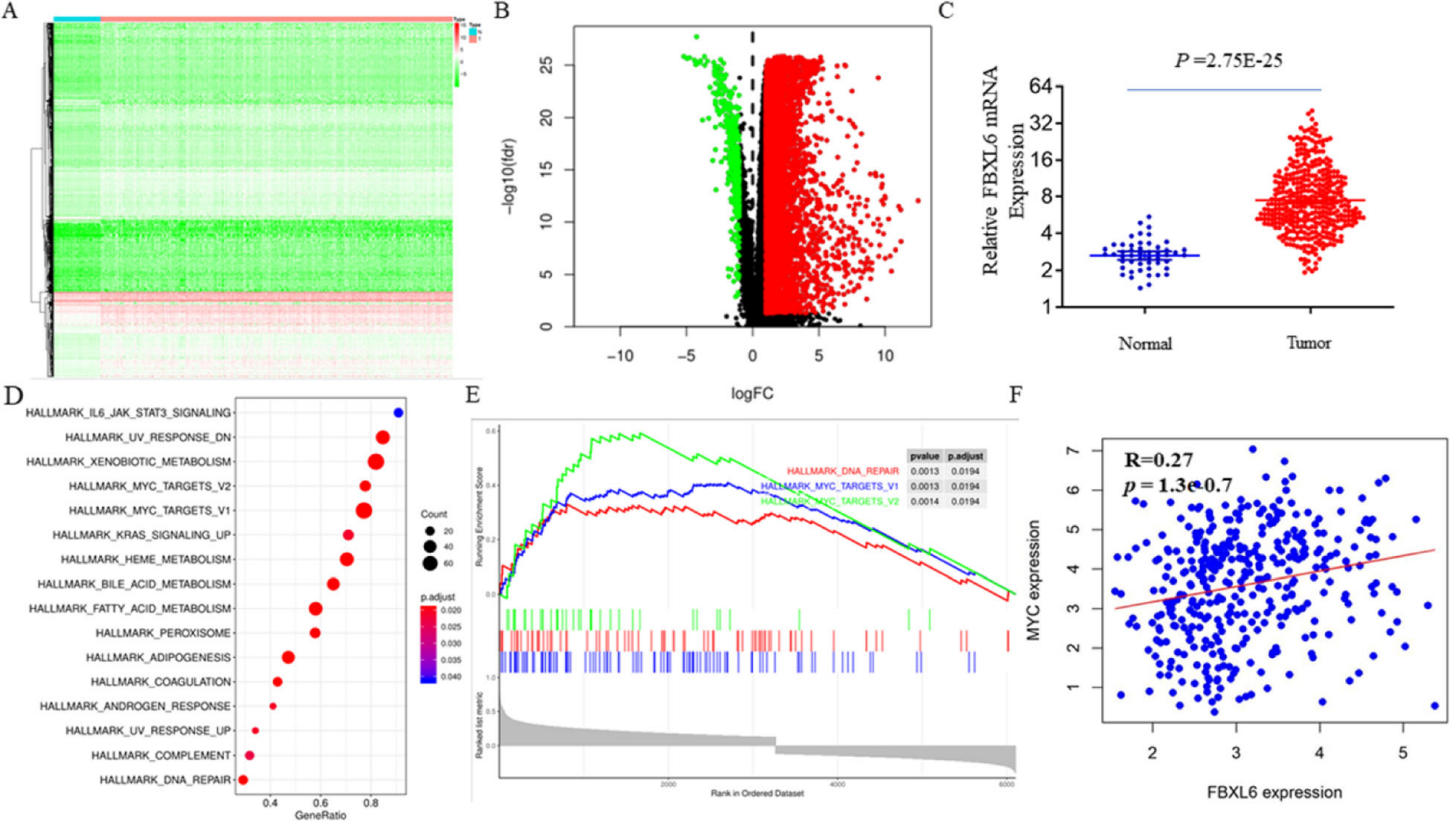
FBXL6 is highly expressed in HCC and associated with the c-MYC pathway. **a** Heatmap demonstrated differentially expressed genes between 374 HCC and 50 non-tumor tissues. **b** Volcano plot demonstrated differentially expressed genes between 374 HCC and 50 non-tumor tissues. Blue dots represent down-regulated genes and red dots represent up-regulated genes. **c** The mRNA expression of FBXL6 between 374 HCC and 50 non-tumor tissues. **d** The GSEA enrichment analysis showed that MYC-targets, bile acid metabolism, fatty acid metabolism and UV response pathways were differentially enriched in patients with high FBXL6 expression. **e** The GSEA enrichment analysis showed that MYC-targets was enriched in 374 HCC patients with high FBXL6 expression. **f** The correlation of the mRNAs of FBXL6 and c-MYC in GEPIA website

To identify key genes involved in the tumorigenesis of HCC, transcriptome RNA-sequencing data of 374 primary HCC samples and 50 non-tumor tissues were downloaded from the TCGA data portal (https://cancergenome.nih.gov/). The Limma R package identified 7667 differentially expressed genes, 7273 up-regulated and 394 down-regulated (Fig. 1a-b). The output of the whole differentially expressed genes was provide in the supplementary Table 1. Among those up-regulated genes, we are particularly interested in F-box proteins, which are usually involved in the development of diverse cancers [23]. For example, the most famous F-box proteins are SKP2, β-TrcP and FBXW7, which are known oncogenes or tumor suppressors [24–26]. We found that the mRNA levels of some F-box proteins were significantly increased in HCC samples when compared with non-tumor tissues, including FBXL18, FBXL16 and FBXL6. FBXL18 has been reported to play an oncogenic role in glioma through promoting K63-linked ubiquitination of Akt [27]. However, the biological function of FBXL16 and FBXL6 proteins are poorly reported. It has been reported that FBXL16 could not interact with Cullin1 to form a SCF complex, indicating an E3 ligase independent function of FBXL16 [28]. Thus, in the current study, we focused on FBXL6, an orphan F-box protein, the expression of which was significantly increased in HCC (*P* = 2.75E-25) (Fig. 1c). In 374 HCC samples, the expression correlation coefficients of FBXL6 and all other genes were calculated using R (Supplementary Table S2), and the Gene Set Enrichment Analysis (GSEA) enrichment analysis was performed using the GSEABase package. We identified many pathways that were significantly enriched, such as MYC-targets, bile acid metabolism, fatty acid metabolism and UV response (Fig. 1d), suggesting that FBXL6 might play a role in these pathways. Notably, given the critical role of c-MYC oncogene in the tumorigenesis of HCC, the enrichment of MYC-target signature suggested a potential regulation of FBXL6 by c-MYC in HCC (Fig. 1e, Supplementary Figure 1). In supporting with this notion, we found that the c-MYC and FBXL6 mRNAs have a notable correlation in liver cancer samples (R = 0.27, *P* = 1.3e-0.7) (Fig. 1f) [29]. Moreover, the expression of FBXL6 was also correlated with many c-MYC target genes including 56.1% MYC activating genes (73/130) and 41.9% MYC repressed genes (13/31) (Supplementary Table S2). Together, these data suggested FBXL6 was highly expressed in HCC samples and associated with the c-MYC pathway.

### FBXL6 exhibits tumor-promoting ability in HCC

**Fig. 2:**
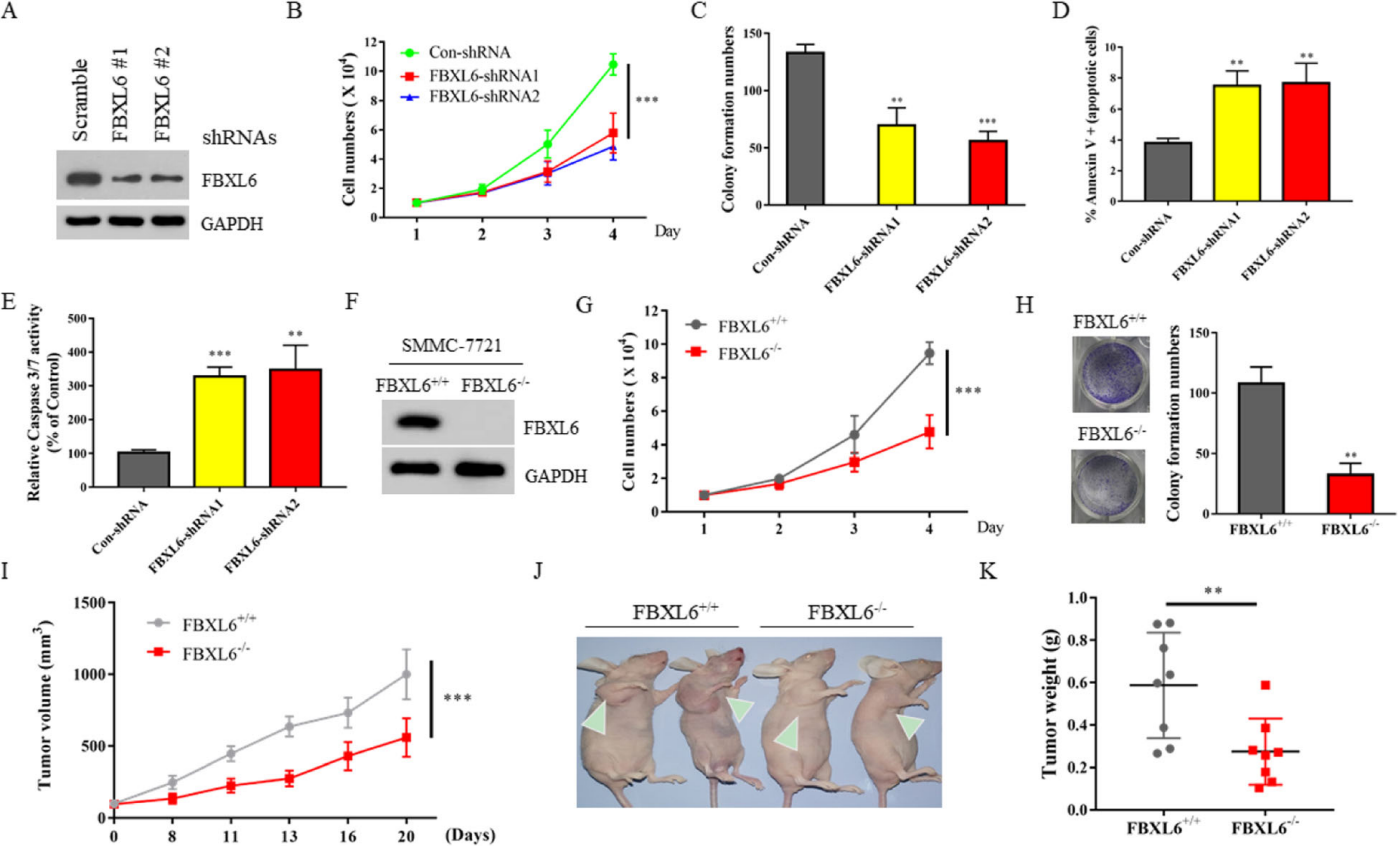
FBXL6 exhibits tumor-promoting ability in HCC. **a** Western blot analysis of the whole cell lysate (WCL) derived from Hep3B cells infected with the indicated shRNA lentiviruses. **b** The cell growth curve of Hep3B cells in (**a**). **c** Clonogenic assay of Hep3B cells in (**a**). **d** Hep3B cells infected with the indicated shRNA lentiviruses were analyzed by FACS with Annexin V-PI assay. The graph represents the percentage of Annexin V positive cells. **e** Caspase3 and Caspase7 activity was measured in Hep3B cells infected with the indicated shRNA lentiviruses. The y axis indicates the caspase3 and caspase7 activity over cell number. The value given for the caspase activity in control-infected cells was set as 100. **f** FBXL6^−/−^ SMMC-7721(KO) cells were generated by CRISPR assay and detected by western blot. **g** The cell growth curve of FBXL6^+/+^and FBXL6^−/−^ SMMC-7721 cells. **h** Clonogenic assay of FBXL6^+/+^and FBXL6^−/−^ SMMC-7721 cells. **i** Each nude mouse was subcutaneously injected with 1 × 10^7^ FBXL6^+/+^ or FBXL6^−/−^ SMMC-7721 cells for about 3 weeks. Tumour growth was measured using a caliper at the indicated times after injection. *n* = 8 for each group. *** *P* < 0.001. **j** The image shows representative tumor-bearing mice for each group. **k** Tumor weights were measured after mice were sacrificed. ** *P* < 0.01

To examine the roles of FBXL6 in growth control, we firstly used two small hairpin RNA (shRNA) constructs to reduce the expression of FBXL6 in Hep3B cells (Fig. 2a). Silencing the expression of FBXL6 caused delayed cell growth and reduced colony formation ability (Fig. 2b-c). Flow cytometry assay showed that the spontaneous apoptosis rate of FBXL6-depelted Hep3B cells was higher than that of control cells (Fig. 2d), and the activities of caspase3 and caspase7 were also enhanced in the absence of FBXL6 (Fig. 2e). To avoid selection of HCC lines that may not accurately reflect the effects of FBXL6 deletion, we also constructed FBXL6 knock out (KO) SMMC-7721 cell line using CRISPR-Cas9 technology, and found that FBXL6 deficiency decreased proliferation and colony formation compared with the control wild type (WT) cells (Fig. 2f-h). Furthermore, we also used nude mice model to investigate whether FBXL6 affected HCC cells proliferation in vivo. Four weeks old BALB/c nude mice were subcutaneously injected with 1 × 10^7^ WT or FBXL6 KO SMMC-7721 cells. We found that knock out of FBXL6 significantly decreased tumor volume and tumor weight compared with WT cells (Fig. 2i-k). Thus, these data indicated that FBXL6 played a critical role in liver cancer cells proliferation both in vitro and in vivo.

### HSP90AA1 is associated with FBXL6

**Fig. 3:**
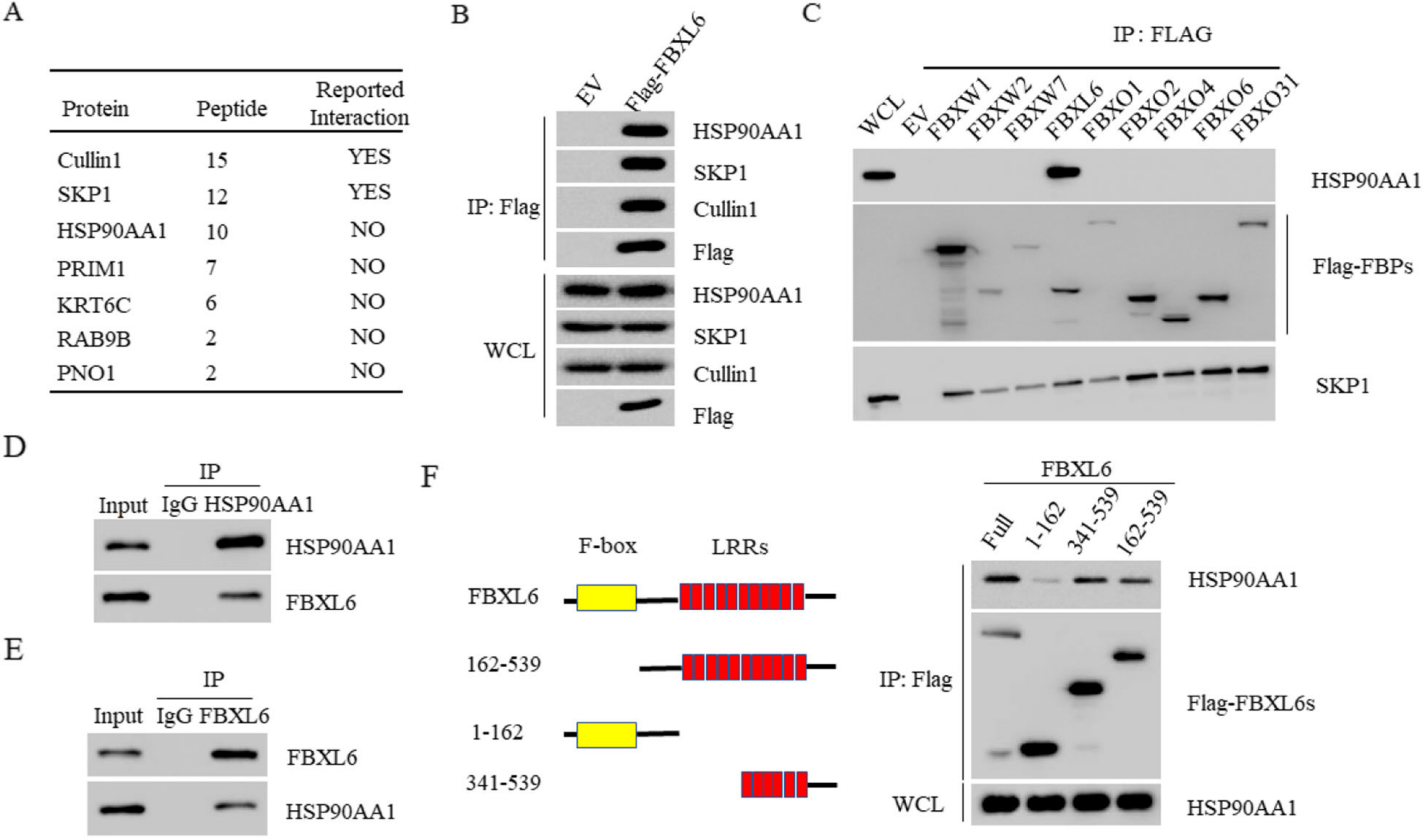
HSP90AA1 is associated with FBXL6. **a** A partial list of proteins identified by mass spectrometry analysis. 293 T cells transfected with Flag-FBXL6 or Flag-Con plasmids were subjected to Flag M2 resin purification. Bound proteins were resolved by SDS-PAGE and stained with Coomassie blue staining, followed by mass spectrometry. **b** 293 T cells transfected with Flag-FBXL6 or Flag-Con plasmids were subjected to Flag M2 purification. Bound proteins were analyzed by immunoblot with indicated antibodies. **c** 293 T cells were infected with retroviruses encoding the indicated Flag-tagged F-box proteins (FBPs) and subjected to Flag M2 resin purification. The immunocomplexes were probed with HSP90AA1, SKP1 and Flag antibodies. Lane 1 shows a whole cell lysate from cells infected with an empty virus (EV). **d** The cell lysate of Hep3B cells were subjected to immunoprecipitation with anti-HSP90AA1 antibody or IgG. The bound proteins were detected by immunoblotting with antibodies against HSP90AA1 and FBXL6. **e** The cell lysate of SMMC-7721 cells were subjected to immunoprecipitation with anti-FBXL6 antibody or IgG. The bound proteins were detected by immunoblotting with antibody against HSP90AA1 and FBXL6. **f** 293 T cells were transfected with the indicated Flag-BRAT1 WT or deletion constructs for 36 h. Cells were harvested and lysed. The WCL was immunoprecipitated by anti-Flag M2 resin and immunoblotted with antibody against HSP90AA1 and Flag

To investigate the molecule mechanism underline the tumor-promoting role of FBXL6, we used the immunoprecipitation/Mass Spectrum (IP/MS) method to identify the interacting proteins of FBXL6. Flag-FBXL6 or Flag-Con plasmids were transfected into 293 T cells and the cell lysates of these cells were subjected to MS analysis after purification by Flag M2 beads. As expected, our MS data analysis identified the known FBXL6 interacting proteins such as Cullin1 and SKP1, suggesting FBXL6 forms a classical SCF E3 ligase complex with both proteins. Importantly, we also identified several unknown new interacting proteins such as HSP90AA1 (Fig. 3a). We then performed western blot assay to confirm our MS data. We found that Cullin1, SKP1 and HSP90AA1 could only be detected in Flag-FBXL6 immunoprecipitate (Fig. 3b). To demonstrate the specificity of this binding, we screened 9 human F-box protein family members. Flag-tagged F-box proteins were expressed into 293 T cells and then immunoprecipitated to evaluate their interaction with endogenous HSP90AA1 protein. Although each F-box protein binds to SKP1, the only F-box protein that binds to HSP90AA1 is FBXL6 (Fig. 3c). The endogenous interaction between FBXL6 and HSP90AA1 was also verified in both Hep3B and SMMC-7721 cells (Fig. 3d-e). FBXL6 is composed of an N-terminal F-box domain and multiple leucine-rich repeat sequences. By protein interaction domain mapping assay, we found that FBXL6 bound to HSP90AA1 via its leucine-rich repeat sequences at its C-terminus (Fig. 3f). Taken together, these data indicated that FBXL6 specifically interacted with HSP90AA1.

### FBXL6 stabilizes HSP90AA1 protein by promoting its K63-ubiquitination

**Fig. 4:**
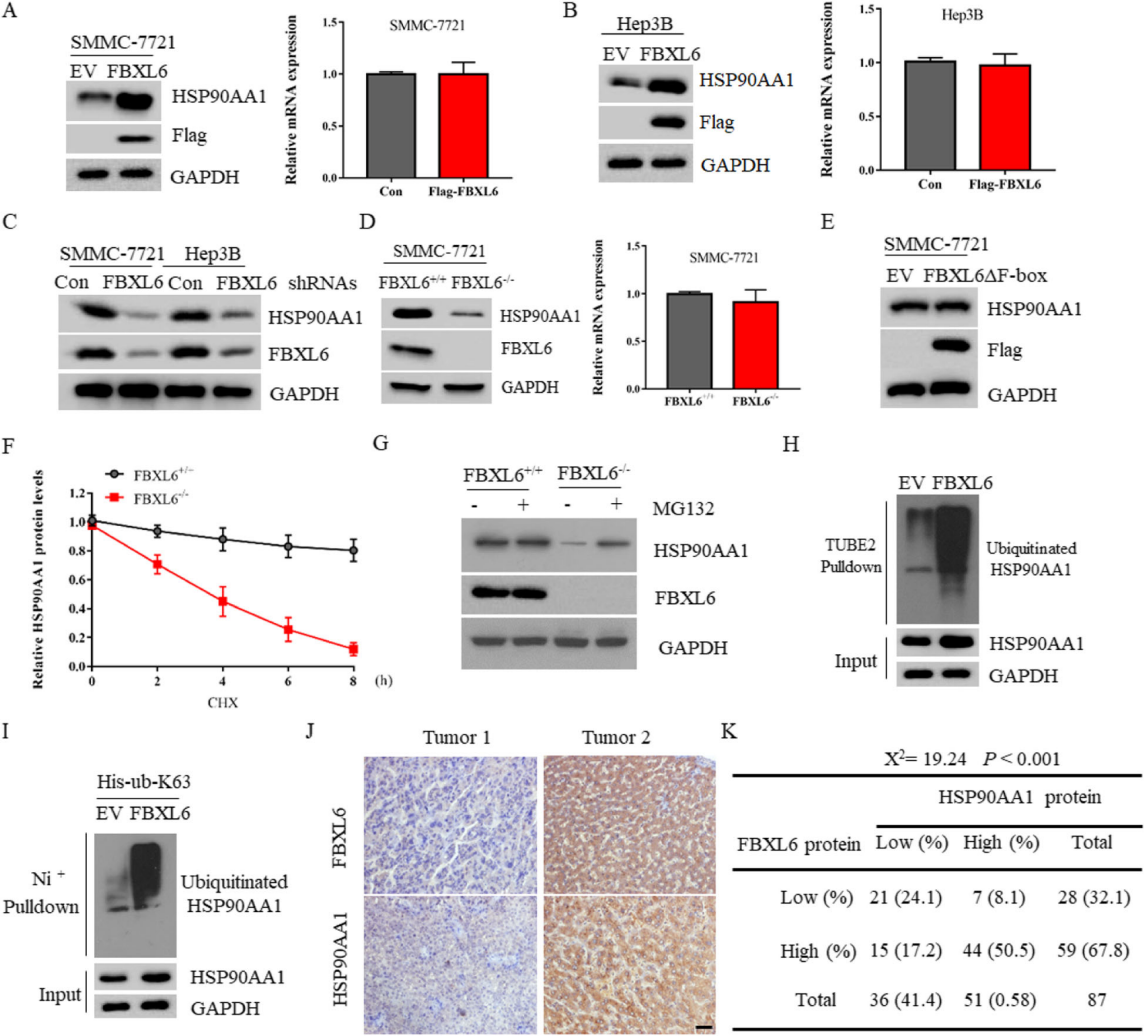
FBXL6 stabilizes HSP90AA1 protein by promoting its K63-ubiquitination. **a** The protein and mRNA levels of HSP90AA1 from SMMC-7721 cells transfected with Flag-Con (EV) or Flag-FBXL6 indicated plasmids were detected by immunoblotting and real-time quantitative PCR, respectively. **b** The protein and mRNA levels of HSP90AA1 from Hep3B cells transfected with indicated plasmids were detected by immunoblotting and real-time quantitative PCR, respectively. **c** Western blot analysis of the WCL derived from SMMC-7721 cells or Hep3B cells infected with the indicated shRNA lentiviruses. **d** The protein and mRNA levels of HSP90AA1 from FBXL6^+/+^and FBXL6^−/−^ SMMC-7721 cells were detected by immunoblotting and real-time quantitative PCR, respectively. **e** Western blot analysis of the WCL derived from SMMC-7721 cells transfected with EV or Flag-FBXL6ΔF-box plasmids. **f** FBXL6^+/+^and FBXL6^−/−^ SMMC-7721 cells were treated with 20 μM cycloheximide (CHX) for the indicated time. The whole cell lysate was immunoblotted with anti-HSP90AA1 antibody. The quantification plot was based on scanning densitometry analysis using the Image J software. Relative protein levels were normalized to FBXL6^−/−^ control group. **g** Western blot analysis of the WCL derived from FBXL6^+/+^and FBXL6^−/−^ SMMC-7721 cells treated with 10 μM MG132 for 6 h. **h** SMMC-7721 cells transfected with EV or Flag-FBXL6 plasmids were harvested and lysed. WCL were immunoprecipitated by Tandem Ubiquitin Binding Entity 2 (TUBE2) resin for ubiquitinated proteins enrichment and immunoblotted as indicated. **i** SMMC-7721 cells were co-transfected Flag-FBXL6 and His-K63-ub for 36 h, cell lysate was subjected to immunoprecipitation by Ni^+^ purification. Immunoprecipitate was detected by immunoblotting using the indicated antibodies. **j** IHC analysis was performed on 87 specimens from HCC patients using anti-FBXL6 and anti-HSP90AA1 antibodies. Representative images of IHC staining of tumors of two HCC patients are presented. **k** The correlation study of FBXL6 and HSP90AA1 protein expression in 87 HCC samples is shown

Typically, F-box proteins usually ubiquitinate substrate proteins and promote their proteasomal degradation [30]. In order to investigate whether FBXL6 can promote the degradation of HSP90AA1, we first overexpressed FBXL6 into SMMC-7721 cells. Interestingly, overexpression of FBXL6 did not reduce HSP90AA1 expression. Instead, it significantly induced the expression of HSP90AA1 protein, without affecting its mRNA level (Fig. 4a). The similar phenomenon was also observed in Hep3B cells (Fig. 4b). On the contrary, silencing the expression of FBXL6 by shRNAs significantly reduced HSP90AA1 expression (Fig. 4c). In agreement, the protein level, but not the mRNA level, of HSP90AA1 was significantly decreased in FBXL6 KO cells compared with WT cells (Fig. 4d). Furthermore, overexpression of an F-box domain-deleted FBXL6 (FBXL6ΔF-box) mutant failed to regulate HSP90AA1 expression (Fig. 4e), suggesting that FBXL6-induced HSP90AA1expression required its E3 ligase activity. To test this possibility, we compared the half-life of HSP90AA1 in HCC cells with or without FBXL6 expression and found that the half-life of HSP90AA1 in FBXL6 KO cells was significantly reduced relative to the WT counterpart (Fig. 4f). Consistently, treatment with the protease inhibitor MG132 restored HSP90AA1 expression in FBXL6 KO cells. (Fig. 4g). However, overexpression of FBXL6 increased the global ubiquitination form of HSP90AA1, suggesting that FBXL6 might promote HSP90AA1 ubiquitination to prevent its degradation (Fig. 4h). Indeed, co-transfected with a His-ubiquitin-K63 plasmid, which coding ubiquitin with only K63 and other lysines were mutated to arginines, showed that FBXL6 significantly promoted HSP90AA1 K63-dependent ubiquitination (Fig. 4i). Since K63-dependent ubiquitination modifications usually do not participate in protein degradation, our data suggest that FBXL6 may promote K63-dependent ubiquitination of HSP90AA1 to stabilize it. In addition, we conducted an IHC analysis to evaluate the potential association between FBXL6 and HSP90AA1 protein in 87 human HCC specimens using anti-FBXL6 and anti-HSP90AA1 antibodies (Fig. 4j). We found that FBXL6 was positively correlated with HSP90AA1 protein in these samples (Fig. 4k, Χ^2^ = 19.24, *P* < 0.001).

### FBXL6 stabilizes c-MYC via HSP90AA1

**Fig. 5:**
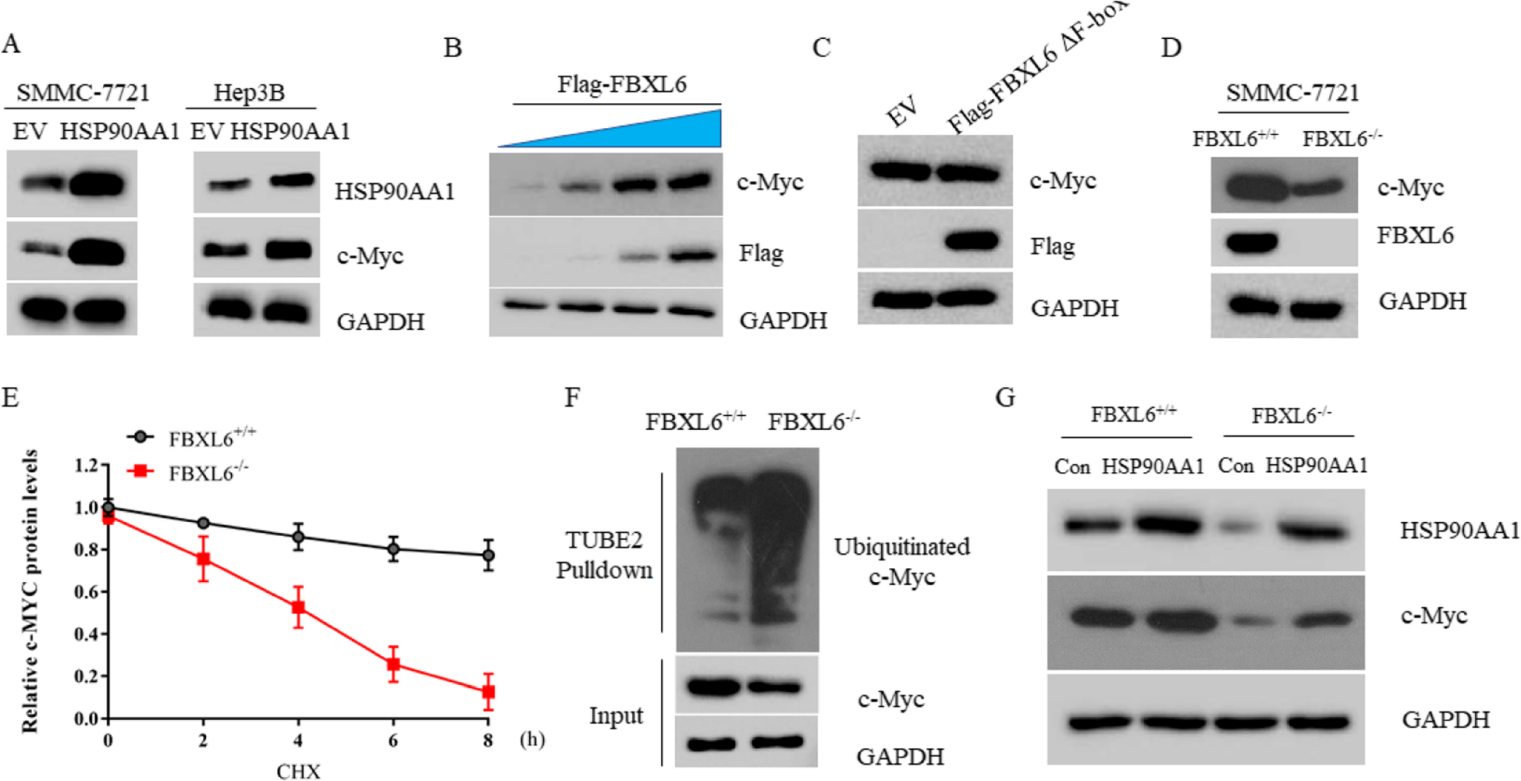
FBXL6 stabilizes c-MYC via HSP90AA1. **a** Western blot analysis of the WCL derived from SMMC-7721 cells or Hep3B cells transfected with EV or HSP90AA1 plasmids. **b** Western blot analysis of the WCL derived from Hep3B transfected with increased dose of Flag-FBXL6. **c** Western blot analysis of the WCL derived from SMMC-7721 cells transfected with EV or Flag-FBXL6ΔF-box plasmids. **d** The protein levels of c-Myc from FBXL6^+/+^and FBXL6^−/−^ SMMC-7721 cells were detected by immunoblotting. **e** FBXL6^+/+^and FBXL6^−/−^ SMMC-7721 cells were treated with 20 μM CHX for the indicated time. The whole cell lysate was immunoblotted with anti-c-Myc antibody. The quantification plot was based on scanning densitometry analysis using the Image J software. Relative protein levels were normalized to FBXL6^−/−^ control group. **f** The WCL from FBXL6^+/+^and FBXL6^−/−^ SMMC-7721 cells were immunoprecipitated by Tandem Ubiquitin Binding Entity 2 (TUBE2) resin for ubiquitinated proteins enrichment and immunoblotted as indicated. **g** FBXL6^+/+^ and FBXL6^−/−^ SMMC-7721 cells transfected with vector control or HSP90AA1 plasmids were subjected to western blot assay with indicated antibodies

HSP90AA1 can exert its tumor-promoting effect by stabilizing c-MYC protein in several cancer types [22, 31]. We found that overexpression of HSP90AA1 in SMMC-7721 and Hep3B also up-regulated c-MYC protein, suggesting c-MYC might be a critical client protein of HSP90AA1 in HCC (Fig. 5a). Moreover, overexpression of FBXL6 promoted the expression of c-MYC in a dose-dependent manner in Hep3B cells (Fig. 5b). However, overexpression of FBXL6ΔF-box mutant could not affect c-MYC expression, suggesting this regulation required the E3 ligase activity of FBXL6 (Fig. 5c). Consistent with this, the expression of c-MYC in FBXL6 KO cells was significantly reduced (Fig. 5d), the half-life of c-MYC protein was shortened (Fig. 5e), and the ubiquitinated form was increased (Fig. 5f). Unsurprisingly, there is no evidence to support the direct binding between FBXL6 and c-MYC protein, suggesting that FBXL6 may regulate c-MYC indirectly. Indeed, overexpression of HSP90AA1 in FBXL6 KO cells partially reversed the FBXL6-induced c-MYC expression (Fig. 5g). Therefore, these data indicated that FBXL6 stabilized c-MYC protein via HSP90AA1.

### C-MYC transcriptional activates FBXL6 in HCC

**Fig. 6:**
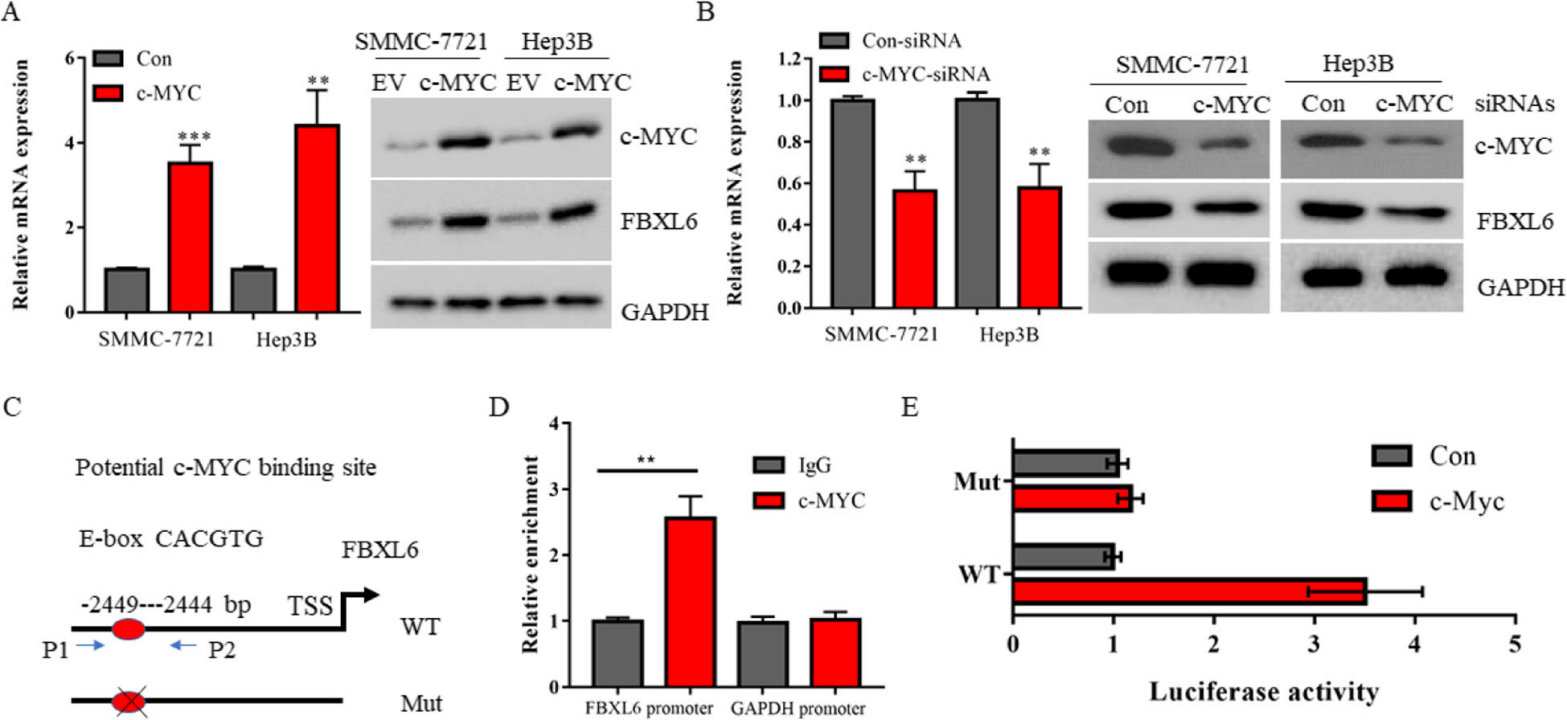
c-MYC transcriptional activates FBXL6 in HCC. **a** The mRNA and protein levels of FBXL6 in SMMC-7721 cells or Hep3B cells transfected with EV or c-Myc plasmid were detected by real-time quantitative PCR and immunoblotting, respectively. **b** The mRNA and protein levels of FBXL6 in SMMC-7721 cells or Hep3B cells infected with the indicated shRNA lentiviruses were detected by real-time quantitative PCR and immunoblotting, respectively. **c** Proximal promoter region of human FBXL6 gene contains a potential binding site of c-Myc. The -3000 bp region from the TSS of FBXL6 was used to scan the potential binding sites of c-MYC. The top one sequence of the software provided was CACGTG, started at 551 and ended at 556. **d** Chromatin immunoprecipitation (ChIP) assays showing representative c-Myc binding to the FBXL6 promoter in Hep3B Cells. Cells were subjected to ChIP assays with anti-IgG or c-Myc antibodies. The promoter of GAPDH was used as negative control. **e** Luciferase reporter assays. HEK293T cells were co-transfected EV or c-Myc plasmids with luciferase reporter plasmids containing wild-type (WT-Luc) or mutant (Mut-Luc) binding site of c-Myc

Bioinformatics analysis reveals that c-MYC and FBXL6 mRNAs have significant correlation in HCC samples (Fig. 1d). Since FBXL6 is an ubiquitin E3 ligase and c-MYC is a classical transcription factor, we hypothesized that c-MYC may regulate the transcription of FBXL6 in HCC. Indeed, overexpression of c-MYC induced the mRNA expression of FBXL6 in both SMMC-7721 and Hep3B cells (Fig. 6a). On the contrast, knockdown of c-MYC by siRNAs inhibited the mRNA expression of FBXL6 (Fig. 6b). The C terminus of c-MYC contains a HLH-LZ domain, which is known to bind to the canonical E-box (CACGTG) to regulate downstream genes expression [32]. We next performed Chromatin immunoprecipitation (ChIP) experiments in Hep3B cells to determine whether c-MYC directly binds to the genomic locus of FBXL6. 3 KB of sequence of the FBXL6 promoter region was then examined for putative c-MYC-binding sites. We used the Jaspar website to locate the exact position of the E box in the promoter of FBXL6 (http://jaspar.genereg.net/). The -3000 bp region from the TSS of FBXL6 was used to scan the potential binding sites of c-MYC. The top one sequence of the software provided was CACGTG, started at 551 and ended at 556 (input 3000 bp), and the score of which was 10.53. Thus, we identified one potential E-box in the FBXL6 promoter region (Fig. 6c). ChIP assay revealed that anti-c-MYC antibody efficiently immunoprecipitated − 2500 bp to − 2300 bp upstream from the transcription start site (TSS) of FBXL6 gene in Hep3B cells (Fig. 6d), suggesting c-MYC directly binds to the promoter region of FBXL6. We also constructed a luciferase reporter vector containing the FBXL6 promoter region of this E-box. The luciferase reporter assay found that c-MYC increased the promoter activity in cells transfected with E-box WT vectors but not in cells with E-box mutant vectors (Fig. 6e). Therefore, our data indicate that FBXL6 is a downstream target gene of c-MYC.

## Discussion

Here, we report that FBXL6 stabilizes HSP90AA1 expression in HCC cells and that FBXL6 expression is correlated with c-MYC expression in HCC tissues, of which a great ratio expresses higher mRNA levels of FBXL6. In line with these observations, we show that genetic inhibition of FBXL6 eliminates HCC cells proliferation in vitro, and thus tumor progression in subcutaneously transplanted HCC mice, indicating a critical role of FBXL6 in the pathogenesis of HCC.

By using IP/MS assay, HSP90AA1 was identified as a putative FBXL6-interacting protein. We further showed that FBXL6 directly interacted with HSP90AA1 via its C-terminus leucine-rich repeat sequences. During the preparation of this manuscript, another group also found that HSP90AA1 was an interacting protein of FBXL6 by high throughput assay [33]. However, the relationship between FBXL6 and HSP90AA1 was still undetermined. Interestingly, unlike the canonical degradation-promoting function of most F-box proteins, we found that FBXL6 ubiquitinates HSP90AA1 to counteract its degradation. Our data further indicate that FBXL6 promotes K63-dependent ubiquitination of HSP90AA1, although we cannot rule out the possibility that FBXL6 may also stimulate other types of ubiquitination, which may help explain this discrepancy. It is possible that FBXL6-mediated HSP90AA1 K63-dependent ubiquitination may antagonize HSP90AA1 K48-dependent ubiquitination promoted by other E3 ligases. Indeed, another F-box protein, FBXL21, has been shown to ubiquitinate cryptochromes to stabilize these proteins [34, 35], suggesting the shared common underline mechanisms of FBXL6 and FBXL21 However, whether FBXL21 also promotes K63-dependent ubiquitination of cryptochromes is still unknown. Therefore, further research is still needed to understand the detailed mechanism of how FBXL6 stabilizes HSP90AA1 protein. The correlation expression of the mRNA levels of FBXL6 and c-MYC promotes us to determine whether FBXL6 is transcriptional regulated by c-MYC. By using ChIP and luciferase assays, we found that c-MYC directly bound to the E-box of FBXL6 promoter region to promote its mRNA expression, suggesting FBXL6 is a downstream target gene of c-MYC in HCC.

## Conclusions

Therefore, our data reveals an unknown positive feedback loop of FBXL6-HSP90AA1-c-MYC axis, and its abnormal regulation may contribute to the occurrence of HCC, and suggests that agents targeting FBXL6 will be beneficial to inhibit HCC.

## Supplementary information

**Additional file 1: Supplementary table 1.** The output of all differentially expressed genes.

**Additional file 2: Supplementary table 2.** The correlations between FBXL6 mRNA expression and MYC target genes.

**Additional file 3: Supplementary Figure 1.** GSEA enrichment analysis of FBXL6 by utilizing the standard GSEA 4.0.1 software.

## Data Availability

Please contact corresponding author for data requests.

## Abbreviations

APC: Anaphase-promoting complex
CRLs: Cullin-based ubiquitin E3 ligases
HCC: Hepatocellular carcinoma
HECT: Homologous to the E6AP carboxyl terminus
HSPs: Heat shock proteins
IP: Immunoprecipitation
LIHC: Liver hepatocellular carcinoma
Limma RING: Linear Models for Microarray and RNA-Seq Data, really interesting new gene
SCF: Skp1/Cullin1/F-box protein
UPS: Ubiquitin (Ub)-proteasome system
