# Supplementary figures and images for: FBXL6 governs c-MYC to promote hepatocellular carcinoma through ubiquitination and stabilization of HSP90AA1
Source: Cell Commun Signal. 2020 Jun 23;18:100. doi: 10.1186/s12964-020-00604-y (PMC7310287; doi:10.1186/s12964-020-00604-y)

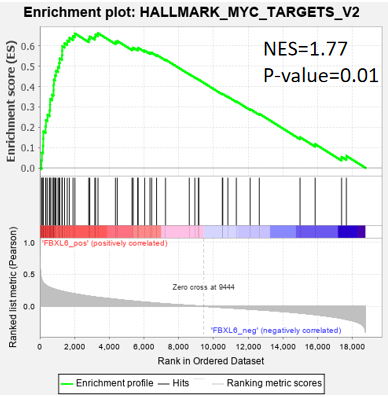

Supplement: Supplementary file 4 — Additional file 3: Supplementary Figure 1. GSEA enrichment analysis of FBXL6 by utilizing the standard GSEA 4.0.1 software. [file 12964_2020_604_MOESM4_ESM.png]
